# Coupling optimization of cell growth cycle and key enzyme membrane localization for enhanced synthesis of high molecular weight heparosan by *Corynebacterium glutamicum*

**DOI:** 10.1186/s40643-025-00899-0

**Published:** 2025-06-17

**Authors:** Jing Yu, Yang Zhang, He Zhang, Zemin Li, Zheng-Jun Li, Tianwei Tan

**Affiliations:** State Key Laboratory of Green Biomanufacturing, Biorefinery Engineering Research Center of the Ministry of Education, National Energy R&D Center for Biorefinery, Beijing University of Chemical Technology, No. 15 of North Three-Ring East Road, Chaoyang District, Beijing, 100029 P.R. China

**Keywords:** Heparosan, *Corynebacterium glutamicum*, Heparosan synthase, High molecular weight

## Abstract

High-molecular weight heparosan (HMW-heparosan) is a member of the glycosaminoglycan family. It possesses various chemical and physical properties suitable for a range of high-quality tissue engineering biomaterials, gels, scaffolds, and drug delivery systems. In this study, the HMW-heparosan biosynthesis pathway was engineered in *Corynebacterium glutamicum* through the introduction of heparosan synthase PmHS2 from *Pasteurella multocida* combined with overexpression of the key genes *ugdA* and *galU*, resulting in the generation of a stable HMW-heparosan-producing strain. Subsequently, to address metabolic flux competition, endogenous glycosyltransferases were systematically deleted to minimize UDP-glucose consumption, leading to a significant increase in HMW-heparosan accumulation. Additionally, cell growth was optimized by overexpressing transcriptional regulators *whcD* and *PnkB*, which was found to improve cell growth while creating an improved intracellular environment for biosynthesis. Notably, the critical enzyme heparosan synthase PmHS2 was relocated to the cell membrane by cell membrane display motifs porB, with its stability and catalytic efficiency being significantly enhanced so that the titer of HMW-heparosan reached 1.40 g/L in shake-flasks. Ultimately, the engineered strain was demonstrated to achieve HMW-heparosan production at 7.02 g/L with an average molecular weight (*M*w) of 801 kDa in 5 L fed-batch bioreactor. These results demonstrate combinatorial optimization of cell factories, especially cell morphology and membrane localization of key enzymes, is efficacious and likely applicable for the production of other biopolymers.

**Graphical Abstract:**

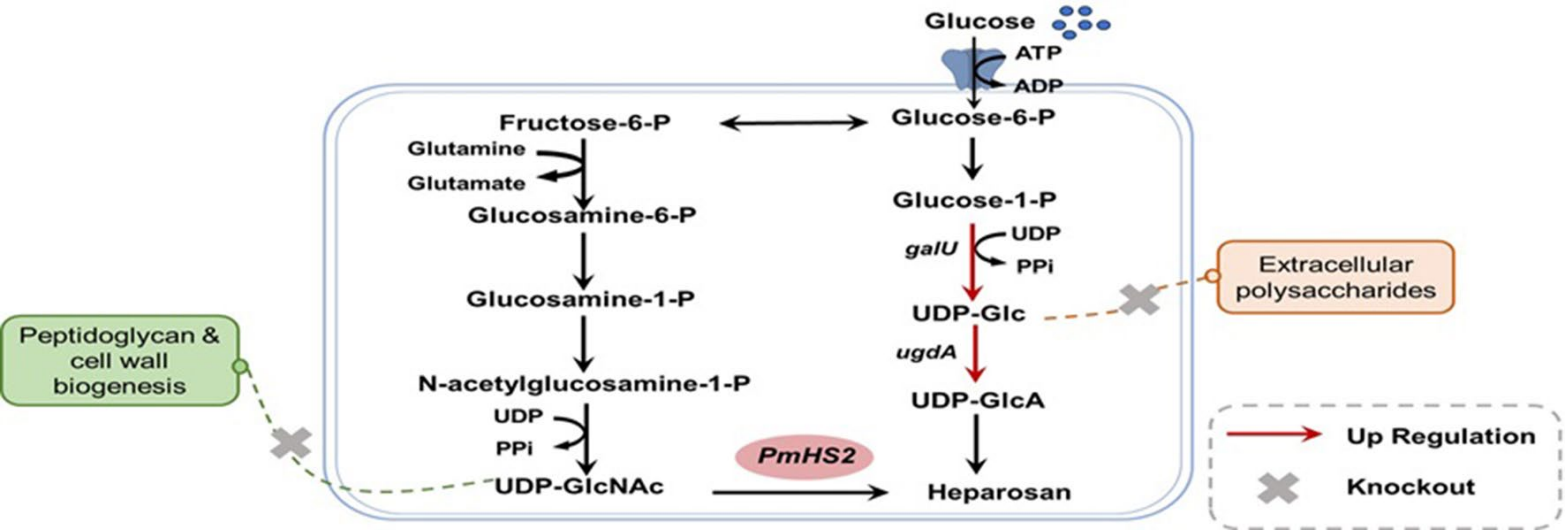

**Supplementary Information:**

The online version contains supplementary material available at 10.1186/s40643-025-00899-0.

## Introduction

Heparin, a widely used anticoagulant drug (Suflita et al. 2015), is primarily employed in surgery to stop vein thrombosis. Heparosan, a natural precursor of heparin and heparan sulfate (HS), belongs to the glycosaminoglycan (GAG) family (Chen et al. 2007). It is composed of repeating disaccharide units formed by β-d-glucuronic acid (GlcA) and *N*-acetyl-α-d-glucosamine (GlcNAc) (Wang et al. 2010). In addition to its use in the synthesis of heparin (Carlsson et al. 2008), heparosan is biocompatible in the human body because it is the endogenous natural precursor in the heparin/heparan sulfate (HS) biosynthetic pathway (Capila and Linhardt 2002). Stretches of heparosan exist in the HS chains found on virtually every human cell thus it is not perceived as a foreign molecule. Recently, heparosan was proven to be a potential alternative to hyaluronic acid in drug carrier design for anticancer therapeutics (Chen et al. 2014). Bacterial capsules composed of heparosan have been reported in *Escherichia coli* K5 (van den Chavaroche and Eggink 2013; Wang et al. 2010), *Escherichia coli* Nissle 1917 (Hu et al. 2022; Yu et al., 2022; Hu et al. 2024) and *Pasteurella multocida* Type D (DeAngelis and White 2002). The biosynthesis of heparosan is regulated in *E. coli* K5 by four genes, *kfiA*, *kfiB*, *kfiC*, and *kfiD*. Genes *kfiA* and *kfiC* are required for polymerization activity, since they are mono-action transferases, respectively encoding for *N*-acetylglucosaminyltransferase and D-glucanosyltransferase (van den Chavaroche and Eggink 2013; Wang et al. 2010; Hu et al. 2022). The synthesis of heparosan in *P. multocida* is regulated by the glycosyltransferases PmHS1 and PmHS2 (Chavaroche et al. 2010; Williams et al. 2019), which exhibit dual activities as both D-glucanosyltransferases and *N*-acetylglucosaminyltransferases. However, the significant risk of pathogenicity, coupled with the presence of endotoxins and exotoxins, limits the utility of heparosan derived from *E. coli* K5 and *P. multocida* in biomedical applications. Therefore, there is growing interest in the development of a safe alternative heparosan production system through the engineering of non-pathogenic microorganisms. Consequently, heterologous expression of *E. coli* K5 or *P. multocida* type D glycosyltransferase genes were attempted in other microorganisms considered safe.

Heparosan molecular weight is an important quality attribute to be considered for biosynthesis of heparin. The molecular weight of microbial-derived heparosan depends on the source of heparosan synthase. Lower molecular weight heparosan of 50–80 kDa (Ly et al. 2011) served as a precursor of heparin is obtained by heparosan synthase from *E. coli* K5. Conversely, heparosan synthase from *P. multocida* is derived for the synthesis of high molecular weight heparosan of 100–800 kDa (Sismey-Ragatz et al. 2007) suitable for biomaterial production. Given the critical influence of polymer size distribution on physicochemical characteristics including viscosity modulation, chain entanglement dynamics, and solubility profiles, the relatively HMW-heparosan produced will be more suitable for producing biomaterials like hydrogels and viscoelastic matrices, while the low sized fractions could serve as heparin precursors in this study. Compared to its homolog PmHS1 (Otto et al. 2012), the PmHS2 glycosyltransferase protein tends to yield more polydisperse products as well as generate polysaccharides through *de novo* synthesis (Lane et al. 2017). Therefore, we selected *Corynebacterium glutamicum* ATCC13032 as the host to construct a recombinant heparosan producer by heterologous expressing PmHS2. Since *C. glutamicum* classified as a “Generally recognized as safe” (GRAS) organism, is relatively rich in glutamine, which can provide amino acids for the HMW-heparosan synthesis process (Li et al. 2023). Meanwhile, the high-density fermentation can be carried out, which is also very favorable for the high titer of target compounds.

Here, we show systematic metabolic engineering of *C. glutamicum* to achieve efficient HMW-heparosan synthesis. Construction of HMW-heparosan synthesis pathway in *C. glutamicum* and overexpression UDP-glucose 6-dehydrogenase and UTP-glucose-1-phosphate uridylyltransferase obtained a stable heparosan-producing strain. Reduced consumption of UDP-glucose by deleting endogenous glycosyltransferases enhanced HMW-heparosan accumulation. Cell growth cycle was regulated to create a favorable HMW-heparosan accumulating environment. Rate-limiting heparosan synthase (PmHS2) was strengthened by relocation to cell membrane. Finally, a high-titer recombinant *C. glutamicum* capable of producing HMW-heparosan was successfully engineered.

## Materials and methods

### Strains and media

*C. glutamicum* ATCC13032 was utilized as the initial host strain for this study, and plasmids construction were executed using *E. coli* Trans10 (TransGen Biotech, Beijing, China). *C. glutamicum* ATCC13032 and its derivatives were cultivated in LBHIS medium (5 g/L NaCl, 10 g/L tryptone, 5 g/L yeast extract, 18.5 g/L brain heart infusion and 91 g/L sorbitol) or LBG medium (5 g/L NaCl, 10 g/L tryptone, 5 g/L yeast extract and 20 g/L glucose). EPO medium (10 g/L NaCl, 10 g/L tryptone, 5 g/L yeast extract, 30 g/L L-glycine and 10 g/L Tween 80) was used for preparing competent cells of *C. glutamicum* and LB-sacB medium (10 g/L NaCl, 10 g/L tryptone, 5 g/L yeast extract and 200 g/L sucrose) was used for gene knockout. *E. coli* was cultured in LB medium (5 g/L NaCl, 10 g/L tryptone and 5 g/L yeast extract).

### Plasmids and strains construction

The plasmids and strains employed in this study are detailed in Tables 1 and 2. The pEC-XK99E containing H36 promoter was used for construction of expression plasmids, and the pK18mobsacB containing Sucrose Lethal allele was selected to construct plasmids for gene deletion (Schäfer et al. 1994). The amplification of DNA fragments was performed using the DNA polymerase PrimeSTAR Max (Takara Bio, Beijing, China). The heterologous genes were synthesized by BGI (BGI, Beijing, China). Plasmid construction was performed using Gibson assembly (Gibson et al. 2009).

**Table 1:** Plamids used or constructed in this study

Plasmids	Description	Sources
pEC-XK99E	Km^R^; *C. glutamicum* cloning vector (PH36 promoter)	Laboratory Storage
pEC-PH36-PmHS2	pEC-XK99E derivative, *PmHS2* gene from *P. multocida*	This study
pEC-PH36-PmHS2-ugdA	pEC-XK99E derivative, PmHS2 gene from *P. multocida*,*ugdA* gene from *Pseudomonas putida*	This study
pEC-PH36-PmHS2-galU	pEC-XK99E derivative, *PmHS2* gene from *P. multocida*,* galU* gene from *C.glutamicum* ATCC 13,032	This study
pEC-PH36-PmHS2-galU-ugdA	pEC-XK99E derivative, *PmHS2* gene from *P. multocida*,*ugdA* gene from *Pseudomonas putida* and *galU* gene from *C.glutamicum* ATCC 13,032	This study
pEC-PH36-PmHS2-galU-ugdA-ptrc-RodA	pEC-PH36-PmHS2-galU-ugdA derivative, *RodA* gene from *C.glutamicum* ATCC 13,032	This study
pEC-PH36-PmHS2-galU-ugdA-ptrc-DivIVA	pEC-PH36-PmHS2-galU-ugdA derivative, *DivIVA* gene from *C.glutamicum* ATCC 13,032	This study
pEC-PH36-PmHS2-galU-ugdA-ptrc-ftsZ	pEC-PH36-PmHS2-galU-ugdA derivative, *ftsZ* gene from *C.glutamicum* ATCC 13,032	This study
pEC-PH36-PmHS2-galU-ugdA-ptrc-PnkB	pEC-PH36-PmHS2-galU-ugdA derivative, *PnkB* gene from *C.glutamicum* ATCC 13,032	This study
pEC-PH36-PmHS2-galU-ugdA-ptrc-PnkC	pEC-PH36-PmHS2-galU-ugdA derivative, *PnkC* gene from *C.glutamicum* ATCC 13,032	This study
pEC-PH36-PmHS2-galU-ugdA-ptrc-whcD	pEC-PH36-PmHS2-galU-ugdA derivative, *whcD* gene from *C.glutamicum* ATCC 13,032	This study
pEC-PH36-CGR-PmHS2-galU-ugdA-ptrc-whcD	pEC-PH36-PmHS2-galU-ugdA-ptrc-whcD derivative, CGR gene from *C.glutamicum* ATCC 13,032	This study
pEC-PH36-Ncgl-PmHS2-galU-ugdA-ptrc-whcD	pEC-PH36-PmHS2-galU-ugdA-ptrc-whcD derivative, Ncgl gene from *C.glutamicum* ATCC 13,032	This study
pEC-PH36-porB-PmHS2-galU-ugdA-ptrc-whcD	pEC-PH36-PmHS2-galU-ugdA-ptrc-whcD derivative, porB gene from *C.glutamicum* ATCC 13,032	This study
pEC-PH36-porC-PmHS2-galU-ugdA-ptrc-whcD	pEC-PH36-PmHS2-galU-ugdA-ptrc-whcD derivative, porC gene from *C.glutamicum* ATCC 13,032	This study
pK18mobsacB	Km^R^; vector for allelic exchange in *C. glutamicum*	Laboratory Storage
pk18-*cgl0350cgl0350*	pK18mobsacB derivative, upstream and downstream of *cgl0350cgl0350*	This study
pk18-*cgl0354*	pK18mobsacB derivative, upstream and downstream of *cgl0354*	This study
pk18-*cgl0363*	pK18mobsacB derivative, upstream and downstream of *cgl0363*	This study
pk18-*cgl0349*	pK18mobsacB derivative, upstream and downstream of *cgl0349*	This study

**Table 2:** Stains used or constructed in this study

strains	Description	Sources
*C.glutamicum* ATCC 13,032	Wild-type strain	Laboratory Storage
cg1	WT derivative, △*cgl0350cgl0350*	This study
cg2	WT derivative, △*cgl0350cgl0350*△*cgl0354*	This study
cg3	WT derivative, △*cgl0350cgl0350*△*cgl0354*△*cgl0363*	This study
cg4	WT derivative, △*cgl0350cgl0350*△*cgl0354*△*cgl0363*△*cgl0349*	This study
cg-PmHS2	WT derivative, included pEC-PH36-PmHS2	This study
cg-galU	WT derivative, included pEC-PH36-PmHS2-galU	This study
cg-ugdA	WT derivative, included pEC-PH36-PmHS2-ugdA	This study
cg-AU	WT derivative, included pEC-PH36-PmHS2-galU-ugdA	This study
cg1-AU	cg1, included pEC-PH36-PmHS2-galU-ugdA	This study
cg2-AU	cg2, included pEC-PH36-PmHS2-galU-ugdA	This study
cg3-AU	cg3, included pEC-PH36-PmHS2-galU-ugdA	This study
cg4-AU	cg4, included pEC-PH36-PmHS2-galU-ugdA	This study
cg3-RodA	cg3, included pEC-PH36-PmHS2-galU-ugdA-ptrc-RodA	This study
cg3-DivIVA	cg3, included pEC-PH36-PmHS2-galU-ugdA-ptrc-DivIVA	This study
cg3-ftsZ	cg3, included pEC-PH36-PmHS2-galU-ugdA-ptrc-ftsZ	This study
cg3-PnkB	cg3, included pEC-PH36-PmHS2-galU-ugdA-ptrc-PnkB	This study
cg3-PnkC	cg3, included pEC-PH36-PmHS2-galU-ugdA-ptrc-PnkC	This study
cg3-whcD	cg3, included pEC-PH36-PmHS2-galU-ugdA-ptrc-whcD	This study
cg3-CGR	cg3, included pEC-PH36-CGR-PmHS2-galU-ugdA-ptrc-whcD	This study
cg3-Ncgl	cg3, included pEC-PH36-Ncgl-PmHS2-galU-ugdA-ptrc-whcD	This study
cg3-porB	cg3, included pEC-PH36-porB-PmHS2-galU-ugdA-ptrc-whcD	This study
cg3-porC	cg3, included pEC-PH36-porC-PmHS2-galU-ugdA-ptrc-whcD	This study

### Gene knockout in *Corynebacterium glutamicum*

The amplified upstream and downstream homologous arms flanking the target gene (approximately 1,000 bp each) were cloned into the suicide plasmid pK18mobsacB. Following this, the recombinant plasmid was electroporated into *C. glutamicum* competent cells, and the transformed cells were centrifuged and plated onto LBHIS plates supplemented with 50 mg/L kanamycin for selection. Colonies exhibiting kanamycin resistance were subsequently screened by PCR for the presence of the *sacB* gene to verify successful integration of the plasmid into the genome through the first homologous recombination event. Positive clones demonstrating both kanamycin resistance and the gene *sacB* amplification were inoculated into 30% sucrose liquid medium and cultured for 24 h (with subculturing for 2–3 generations if required) until turbidity developed, after which they were streaked onto 20% sucrose solid medium to promote counterselection. Single colonies from sucrose plates were analyzed for the *sacB* gene, and those failing to amplify the *sacB* gene—indicative of a second homologous recombination event leading to plasmid excision and target gene deletion were further validated by secondary PCR. Finally, confirmed knockout strains were expanded in LBHIS liquid medium, purified through streaking on LBHIS plates, and cryopreserved for downstream applications following comprehensive verification.

### Strain cultivation and fed-batch fermentation

The fermentation medium consisted of the following components: glucose (60 g/L), corn steep powder (20 g/L), KH_2_PO_4_ (1 g/L), K_2_HPO_4_ (1 g/L), urea (5 g/L) and MgSO_4_ (1.2 g/L). The shake-flask experiments were conducted in triplicate using 250 mL sealed shake flasks containing 50 mL of fermentation media with an initial OD_600_ of 0.1. Shake-flask fermentations were conducted at 30℃, 200 rpm for 48 h.

For fed-batch fermentations, the final pH of the seed and fermentation medium was adjusted to 7.0. A total of 50 mg/L kanamycin sulfate was added to all media for detection of transformants or for recombinant culture. The seed culture was carried out in LBG media at 30℃ with shacking at 200 rpm for 12–16 h. The initial inoculation OD_600_ in the 5 L bioreactor was 1.5 and the pH was adjusted to 7.0 by adding 6.5% sulfuric acid and 25% ammonia. The temperature was maintained at 30℃. The addition rate was adjusted based on the changes in glucose concentration in the fermentation medium.

### Measurement of biomass and glucose concentration

Cell density was determined by measuring the turbidity of the culture medium at 600 nm using a spectrophotometer (Thermo Scientific, Waltham, MA) and the growth rate was calculated from the cell density data.

The glucose concentration was determined by UltiMate 3000 HPLC (Thermo Scientific, Waltham, MA) using the following apparatus and operating conditions: Bio-Rad Aminex HPX-87 H column (Bio-Rad Laboratories, Hercules, CA) with RID and UV detectors; column temperature of 65℃; and 0.6 mL/min of 5 mM sulfuric acid as mobile phase.

### Heparosan titer assay

The fermentation broth was centrifuged to separate the supernatant from the cell pellet. The cell pellet was then resuspended in deionized water and disrupted using a high-pressure homogenizer at a pressure of 1500 bar for 5 min. The resulting supernatant was collected via centrifugation. To precipitate heparosan, 3% NaCl was added to the supernatant, followed by the addition of three volumes of pre-cooled absolute ethanol. This mixture was allowed to precipitate at 4 °C for 2 h. After centrifugation, the supernatant was discarded, and the heparosan was redissolved in distilled water.

The carbazole-sulfuric acid method is an internationally recognized technique for quantifying heparosan by indirectly measuring glucuronic acid content. This method has become the preferred approach for determining uronic acid levels, with uronic acid comprising 46.56% of heparosan. To quantify heparosan, 1 mL of the heparosan sample was mixed with 5 mL of sulfuric acid solution. The mixture was boiled for 10 min and then cooled to room temperature. Following this, 200 µL of carbazole reagent was added, and the mixture was boiled for an additional 15 min. The absorbance was measured at 530 nm, and the heparosan content was calculated based on the standard curve.

### Extracellular polysaccharide analysis in *C. glutamicum*

Strains *C. glutamicum*, cg1-AU, cg2-AU, and cg3-AU were cultured at 28 °C for 48 h. Bacterial cells were subjected to mechanical disruption *via* glass bead vertexing (200 rpm, 1 h, room temperature) to liberate surface-associated materials, including extracellular polysaccharides (Puech et al. 2001a, b). Cellular debris was removed by centrifugation (4,000 rpm, 20 min), and the resulting supernatants were pooled for polysaccharide hydrolysis using 2 M trifluoroacetic acid (110 °C, 2 h). Hydrolysates containing reducing sugars were dried under vacuum at 65 °C for 2 h. To derivatize the sugars, the dried residues were reacted with 1-phenyl-3-methyl-5-pyrazolone (PMP) in a NaOH-methanol solution (70 °C, 60 min) to form sugar-PMP conjugates (Wang et al. 2018).

Sugar composition analysis was performed on an Agilent 1200 series HPLC system equipped with a SHISEIDO CAPCELL PAK C18 column (4.6 mm × 250 mm, 5 μm particle size). Separation was achieved using an isocratic mobile phase comprising 0.1 M KH2PO4 (pH 6.8, 82% v/v) and acetonitrile (18% v/v) at a flow rate of 1 mL/min. PMP-sugar derivatives were detected by UV absorbance at 245 nm.

### Heparosan purification and heparosan weight-average molecular weight

Separation and purification of heparosan using AKTA. Firstly, the packed DEAE-Sephacel was subjected to a column loading operation under controlled pressure. After the column was loaded, the column was equilibrated with 5 column volumes buffer A (50 mM NaCl, 50 mM NaAc, pH 4.5), and then the sample was loaded. After the sample loading was completed, the column was rinsed with 10 times the column volume of buffer A to wash away the impurities that were not adsorbed with the packing material, and then eluted with buffer B (1.5 M NaCl, 50 mM NaAc, pH 4.5) to elute the adsorbed heparosan samples, and the samples were collected according to the absorption peaks at 205 nm in the process of elution. Finally, the eluate was desalted with a dialysis bag, and the desalted eluate was lyophilized with the freeze dryer to obtain the purified sample.

The molecular weight distribution was analyzed using a Waters 1515 gel permeation chromatography (GPC) system equipped with a refractive index detector and coupled in series with two columns: Waters Ultrahydrogel 250 (7.8 × 300 mm) and Waters Ultrahydrogel 2000 (7.8 × 300 mm). Chromatographic separation was conducted using a mobile phase of 0.1 M sodium nitrate (NaNO₃) at a flow rate of 0.5 mL/min, with both the column and detector temperatures maintained at 35 °C. Samples (50 µL injection volume) were run for 60 min per analysis. For preparation, approximately 5 mg of the polysaccharide dissolved in 1 mL of ultrapure water, and subsequently filtered through a 0.22 μm membrane to remove particulate matter. Data acquisition and molecular weight calculations were performed using Breeze 2 software, which analyzed retention times against a calibration curve to determine weight-average molecular weight (*M*w) and polydispersity index (PDI).

### Transmission electron microscopy

One cubic millimeter of wet cells was mixed with 1.0 ml of 2.5% glutaraldehyde solution and suspended in a centrifuge tube for more than 6 h. Then, the cells were washed three times with 0.1 M phosphate-buffered solution (PBS) and fixed with 4% paraformaldehyde fixative for 2 h. After fixation, the cells were washed with 0.1 M PBS for three times again, and it was dehydrated with gradient ethanol before freeze-drying. The prepared cell samples were characterized by transmission electron microscopy (FEI Tecnai Spirit G2 BioTWIN, FEI) and field emission electron microscopy (GeminiSEM 500, ZEISS).

## Results and discussions

### Constructing HMW-heparosan synthesis pathway in *C. glutamicum* ATCC13032

**Fig. 1:**
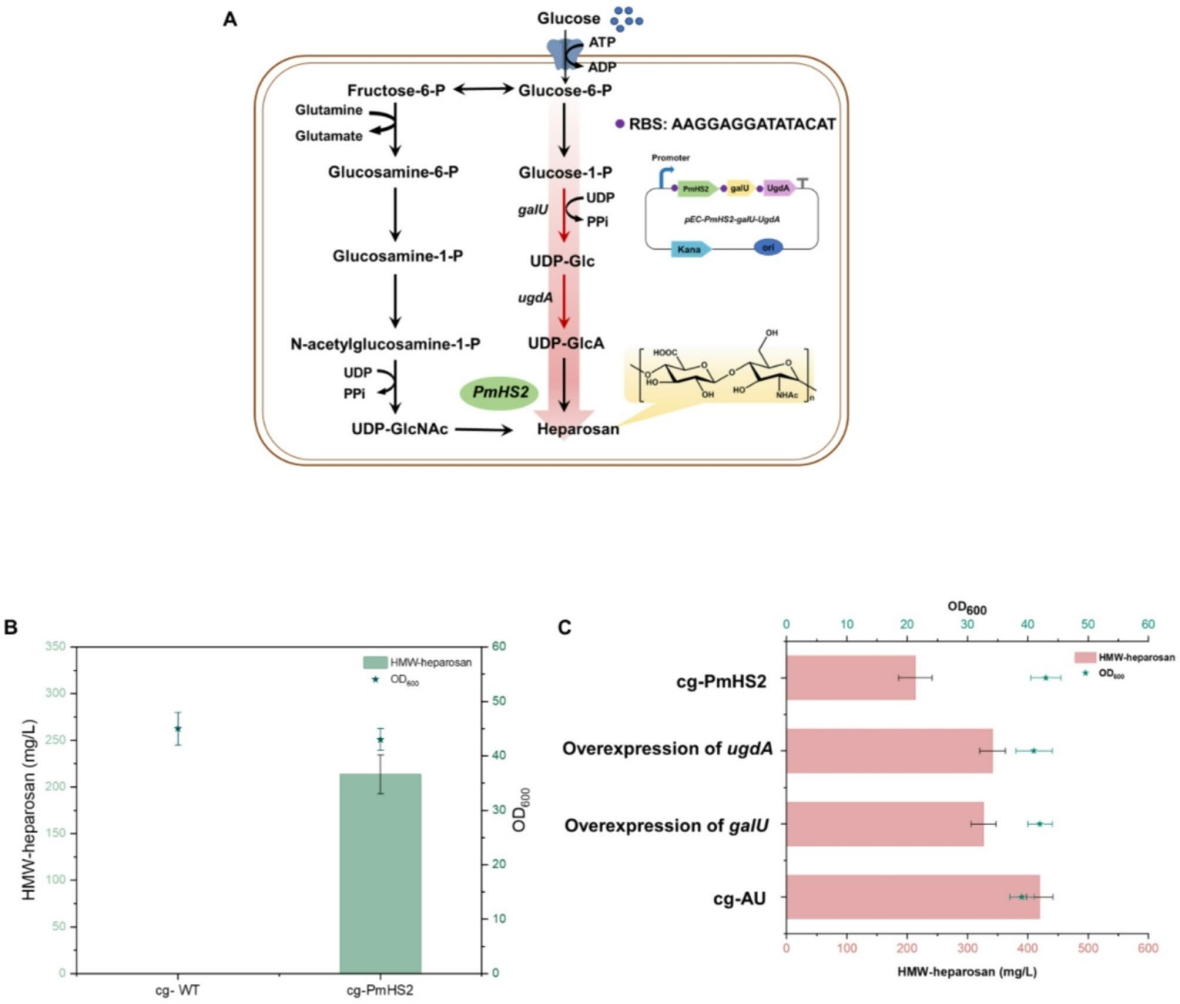
(**A**) Engineering *C. glutamicum* for HMW-heparosan biosynthesis. Heparosan chains are elongated by heparosan synthase (PmHS2) from two building blocks: UDP-GlcA (uridine diphosphate glucuronate) and UDP-GlcNAc (uridine diphosphate *N-*acetylglucosamine). *galU*, glucose-1-phosphate uridylyltransferase; *ugdA*, UDP-glucose 6‐dehydrogenase. (**B**) HMW-heparosan production in engineered strain cg-PmHS2 expressing heterologous PmHS2. (**C**) The effect of individual overexpression of *galU* or *ugdA* versus their co-expression on HMW-heparosan production and cell growth (OD_600_)

In wild-type *C. glutamicum* ATCC 13,032 lacks endogenous heparosan synthase, it inherently produces the two key metabolic precursors required for heparosan biosynthesis: UDP-GlcA and UDP-GlcNAc. In this study, the bifunctional heparosan synthases PmHS2 from *P. multocida* (DeAngelis and White 2002; May et al. 2001; Williams et al. 2019) heterologous expressed in *C. glutamicum* and the HMW-heparosan biosynthesis pathway was constructed (Fig. 1A). Successful expression of the 75.3 kDa PmHS2 protein was confirmed by SDS-PAGE analysis (Figure S1), with subsequent shake flask cultivations yielding 213.6 mg/L HMW-heparosan over 48 h (Fig. 1B). To verify structural fidelity of the biosynthesized product, purified samples underwent comprehensive NMR characterization. The ¹H-NMR spectrum revealed characteristic heparosan resonances at δ 5.32 (s, 1 H, anomeric proton of GlcNAc), 4.42 (s, 1 H, GlcA anomeric proton), 3.32 (s, 2 H, GlcA anomeric proton), and 1.97 (s, CH3, anomeric proton of GlcNAc) - all showing excellent correspondence with commercial heparosan standards (Figure S2). Meanwhile, the purified bioproduct was enzymatically digested with heparinase III to produce an unsaturated disaccharide. The product disaccharide showed the same retention time as the known ΔUA-GlcNAc standard by SAX-HPLC analysis (Figure S3).

### Identifying metabolic bottleneck to improve HMW-heparosan production

A comprehensive analysis of UDP precursor biosynthetic pathways is essential for the development of an efficient microbial cell factory for HMW-heparosan production. Identifying metabolic bottlenecks during UDP precursor biosynthesis is crucial, as these bottlenecks can significantly impede heparosan production. UDP-glucose dehydrogenase is regarded as a potential rate-limiting enzyme, responsible for catalyzing the oxidation of UDP-glucose to UDP-GlcA (Chen et al. 2017; Roman et al. 2003). Additionally, another critical enzyme, glucose-1-phosphate uridylyltransferase provides the precursor UDP-glucose for the rate-limiting step in heparosan biosynthesis (Roman et al. 2003; Wang et al. 2011). Addressing this challenge entails overcoming the rate-limiting step in precursor biosynthesis, which can be accomplished by enhancing the expression of the rate-limiting enzyme and co-expressing the rate-limiting gene alongside other genes in the metabolic pathway (Jin et al. 2016; Hu et al., 2022; Zhang et al. 2018).

When overexpression of *ugdA* increased the titer of HMW-heparosan to 341.5 mg/L, which was 59.3% higher than that of cg-PmHS2. Overexpression of *galU* increased the titer of HMW-heparosan to 326.4 mg/L, a 52.8% increase over cg-PmHS2 (Fig. 1B). More importantly, a breakthrough increased in HMW-heparosan titer to 426.7 mg/L (nearly 100% increase over cg-PmHS2) was achieved when the *ugdA-galU* dual-gene co-expression strategy was employed (Fig. 1C). These results not only confirmed that UDP-GlcA is a key limiting factor for HMW-heparosan synthesis, but also provided a clear target for metabolic engineering modification. A systematic understanding of metabolic bottlenecks in UDP-precursor biosynthesis is critical for developing efficient microbial cell factories dedicated to heparosan synthesis. Notably, UDP-glucose dehydrogenase (encoded by *ugdA*) has been established as the key rate-limiting enzyme, catalyzing the irreversible conversion of UDP-glucose to UDP-GlcA (Chen et al. 2017; Roman et al. 2003). Additionally, glucose-1-phosphate uridylyltransferase (encoded by *galU*) plays an indispensable upstream role by generating the essential UDP-glucose substrate required for UDP-glucose dehydrogenase activity (Roman et al. 2003; Wang et al. 2011).

To alleviate these metabolic constraints and enhance heparosan production, overexpression of the gene *ugdA* increased heparosan titers to 341.5 mg/L, representing a 59.3% improvement over the strain cg-PmHS2. Similarly, the gene *galU* overexpression elevated heparosan production to 326.4 mg/L, a 52.8% increase compared to the cg-PmHS2. Most significantly, synergistic co-expression of *galU* and *ugdA* (encoding UDP-glucose dehydrogenase) generated a compounding effect, achieving a heparosan titer of 426.7 mg/L (Fig. 1B). These findings collectively demonstrate that targeted amplification of rate-limiting enzymes coupled with co-expression of critical pathway genes effectively alleviated flux constraints in UDP-precursor biosynthesis, thereby achieving a 2.0-fold enhancement in heparosan production.

### Enhancing of HMW-heparosan production by attenuating the biosynthesis of extracellular polysaccharides

**Fig. 2:**
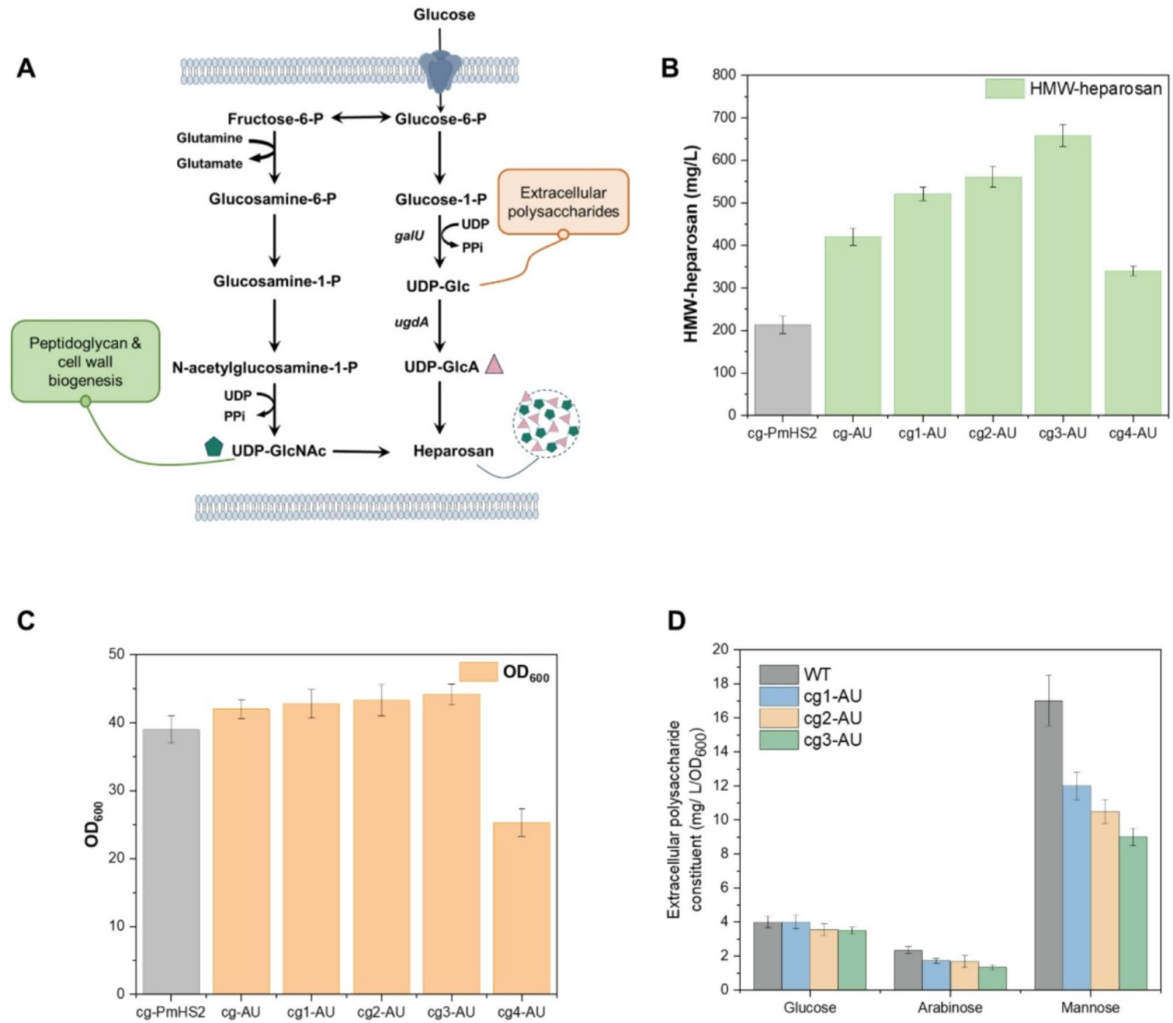
Attenuation of extracellular polysaccharide biosynthesis to enhance HMW-heparosan production. (**A**) HMW-heparosan biosynthesis in *C. glutamicum* and associated extracellular polysaccharide pathways. (**B**) The effect of deleting Δ*cgl0350* Δ*cgl0354* Δ*cgl0363* and Δ*cgl0349* on HMW-heparosan production in (**C**)* glutamicum*. The strain cg-AU, cg1-AU (Δ*cgl0350*), cg2-AU (Δ*cgl0350* Δ*cgl0354*), cg3-AU (Δ*cgl0350* Δ*cgl0354* Δ*cgl0363*), and cg4-AU (Δ*cgl0350* Δ*cgl0354*Δ*cgl0363* Δ*cgl0349*). C. The effect of deleting Δ*cgl0350* Δ*cgl0354* Δ*cgl0363* and Δ*cgl0349* on cell growth (OD_600_). (**D**) Sugar constituent of cell-surface polysaccharides isolated from *C. glutamicum* (WT), cg1-AU, cg2-AU and cg3-AU

We then investigated metabolic pathways that compete with HMW-heparosan synthesis, focusing on the biosynthetic processes of extracellular polysaccharides, which consume critical intermediates UDP-glucose (Donot et al. 2012), a key precursor for both UDP-GlcA and HMW-heparosan synthesis (Fig. 2A). The corynebacterial cell wall contains a peptidoglycan attached arabinogalactan structure, which is enveloped by mycolic acid and an outer layer of extracellular polysaccharides, proteins and lipids (Puech et al. 2001a, b). The main components of corynebacterial extracellular polysaccharides (also considered as capsular polysaccharides or cell-surface polysaccharides) are arabinomannan, mannan and glucan (Jackson 2025). Previous studies revealed that deleting *cgl0350* and *cgl0354* significantly reduced mannose and arabinose levels in extracellular polysaccharide hydrolysis, suggesting these genes encode mannosyltransferase and arabinosyltransferase (Wang et al., 2020).

To redirect metabolic flux toward HMW-heparosan synthesis, we engineered knockout strains cg1 (deletion of *cgl0350*), cg2 (deletion of *cgl0350* and *cgl0354*), cg3 (deletion of *cgl0350*, *cgl0354* and *cgl0363*) and cg4 (deletion of *cgl0350*, *cgl0354*, *cgl0363* and *cgl0349*). Deletion of *cgl0350*, *cgl0354*, or *cgl0363* did not affect cell growth but reduced intracellular mannose and arabinogalactan. The strain cg1-AU reduced the mannose and arabinose concentrations released from hydrolyzed extracellular polysaccharides by 29.4% and 26.4%, respectively. Similarly, individual knockout of *cgl0354* resulted in a 38.2% decrease in mannose content within extracellular polysaccharides, while arabinose levels remained unaffected. Notably, dual knockout of *cgl0354* and *cgl0363* in strain cg3-AU led to more pronounced reductions, with mannose and arabinose concentrations decreasing by 47.06% and 43.0%, respectively. The engineered strain cg1-AU achieved the HMW-heparosan titer of 521.2 mg/L (Fig. 2B). Sequential deletions in strains cg2-AU and cg3-AU further elevated titers to 561.5 mg/L and 658.2 mg/L (Fig. 2B), respectively. The cg3-AU strain demonstrated a nearly 3-fold increase in HMW-heparosan production compared to the control strain cg-PmHS2. These results demonstrate that blocking competing polysaccharide synthesis pathways effectively redirects carbon flux toward HMW-heparosan production. In contrast, disruption of *cgl0349* markedly impaired cellular growth, with a 35.90% reduction in biomass accumulation as measured by OD_600_ (Fig. 2C). This observation strongly suggests an indispensable role of *cgl0349* in maintaining cell wall structural integrity, precluding its utility as a metabolic engineering target.

### Improving HMW-heparosan production by regulation cell growth cycle

**Fig. 3:**
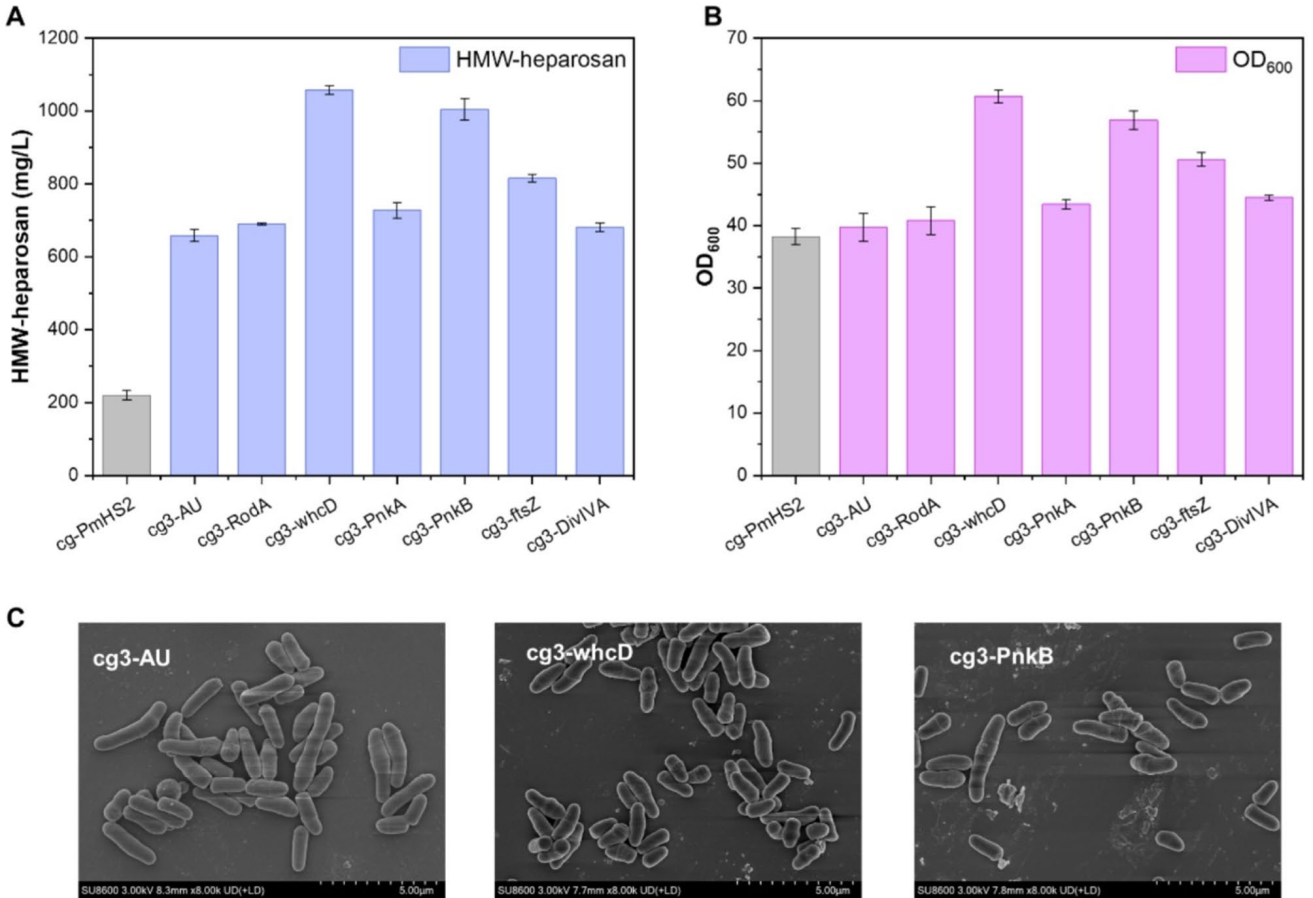
Improving HMW-heparosan production by regulation cell growth cycle (**A**) The effect of HMW-heparosan production by overexpression of genes associated with changes in cell morphology. (**B**) The effect of cell growth (OD_600_) by overexpression of genes associated with changes in cell morphology. (**C**) The cell morphology of cg3-AU (wild type), cg3-whcD and cg3-PnkB

HMW-heparosan biosynthesis faces a critical challenge in *C. glutamicum* that the absence of dedicated ABC transporters leads to intracellular polymer accumulation (Corbett and Roberts 2008; Cuthbertson et al. 2010a, b), causing substantial spatial constraints. To address this limitation, we implemented a cell cycle regulation strategy designed to create an optimized intracellular environment for HMW-heparosan storage. Several genes associated with the cell growth and division cycle of *C. glutamicum* were selected, including *DivIVA*, *RodA*, *ftsZ*,* pknA*, *pknB*, and *whcD*. Among them, *DivIVA* and *RodA* are responsible for cell polar growth (Letek et al. 2008; Sieger et al. 2013). *FtsZ* plays a key role in recruiting other proteins that facilitate the invagination of plasma membrane, leading to cell division (Ramos et al. 2005). This process is s also regulated by various transcriptional regulators including *PknA*, *PknB*, and *whcD* (Fiuza et al. 2008; Lee et al. 2017). Individual overexpression of these genes revealed distinct phenotypic effects. Notably, overexpression of *PknB* and *whcD* resulted in an increase in the cell density (OD_600_) by 1.4 and 1.5 folds (Fig. 3B)., respectively, compared to the control strain. However, no significant changes in cell size were observed (Fig. 3C). It demonstrates that overexpression of these genes enhances HMW-heparosan synthesis through enhanced cell growth rather than changes in cell size. Therefore, the HMW-heparosan titer of the cg3-whcD strain was 1057.2 mg/L (Fig. 3A) cultured in shake flasks for 48 h.

### Increasing HMW-heparosan production by membrane-located expression of PmHS2

The enhancement of PmHS2 activity and stability is crucial for HMW-heparosan synthesis in *C. glutamicum.* The re-localization of PmHS2 to the cell membrane created a microenvironment that minimized interference from cytoplasmic components, similar to enzyme entrapment immobilization. For example, in *Bacillus thuringiensis*, relocating chitinase to the cell membrane by using an anchoring motif enhanced both its activity and stability (Tang et al. 2017). A similar strategy improved xylanase activity in *E. coli* through membrane relocation using anchoring motifs (Chen et al. 2012). Similarly, relocating PHA synthase to the cell membrane in *C. glutamicum* significantly increased polyhydroxyalkanoate (PHA) titer (Jin et al. 2022).

**Fig. 4:**
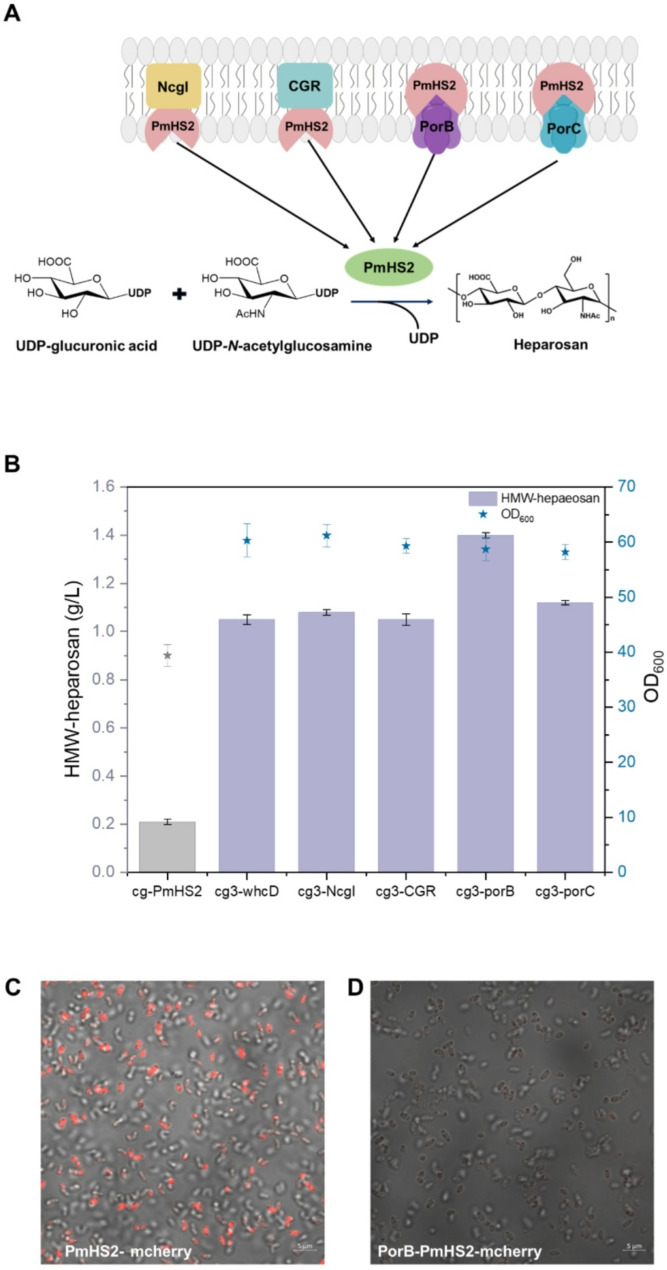
Increasing HMW-heparosan production by membrane-located expression of PmHS2. (**A**) Diagram of membrane-located expression of heparosan synthase (PmHS2). (**B**) The effect of HMW-heparosan production and cell growth (OD_600_) by membrane-located expression of PmHS2. (**C**) The CLSM result of PmHS2-mchrerry. (**D**) The CLSM result of PorB-PmHS2-mcherry

In the present study, PmHS2 was similarly relocated to the cell membrane of *C. glutamicum* to augment its activity and stability. This relocation provided a microenvironment for PmHS2 by embedding it as a carrier within the cell membrane, thereby mitigating the influence of cytoplasmic components, akin to enzyme immobilization. Furthermore, the localization of PmHS2 reduced the dilutional effect of the protein by fixing it to the cell membrane, which may also contribute to increased stability. Here, the secreted signal peptides Ncgl (Yim et al.,^2016^) and CGR (Zhang et al. 2015) and cell membrane display motifs porB and porC (Tateno et al. 2009) were utilized as membrane anchors. The N-terminus of PmHS2 was fused to the C-terminus of Ncgl, CGR, porB, and porC, respectively, to facilitate the membrane-localized expression of PmHS2 (Fig. 4A). Notably, the use of porB for localizing PmHS2 was found to be more favorable for HMW-heparosan accumulation. The production of HMW-heparosan increased to 1.40 g/L (Fig. 4B), nearly 7-fold increasement compared to the control strain cg-PmHS2. When we fused porB at the N-terminal end of PmHS2 and mcherry at the C-terminal end, the CLSM results analyzed that the fluorescence of the proteins on the cell surface was weakened as compared to PmHS2-mcherry (Fig. 4C, D). PorB-PmHS2 displayed on the surface of *C. glutamicum* (Fig. 4D) because fluorescent proteins are readily reduced by other substances in the extracellular environment and thus on the cell surface loses fluorescence (Yang et al. 1996).

**Fig. 5:**
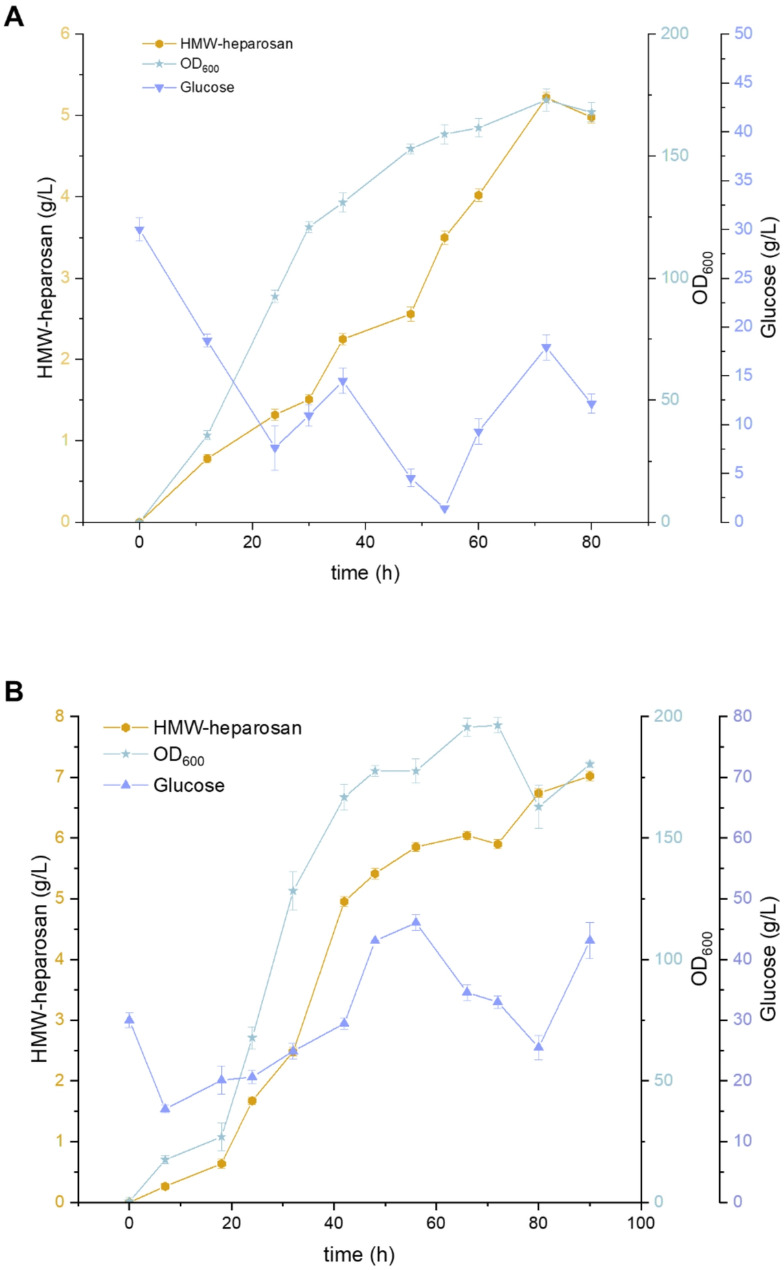
Fed-batch fermentation in a 5 L bioreactor. **A**. Fed-batch fermentation at a constant stirring speed of 500 rpm. **B**, Fed-batch fermentation under two-phase stirring speed control (300 rpm to 500 rpm)

To assess the scalability of HMW-heparosan production, fed-batch experiments were conducted in 5 L bioreactors using the strain cg3-porB. We maintained a rotational speed of 500 rpm, resulting in the highest HMW-heparosan titer of 5.22 g/L (Fig. 5A). However, during the initial phase, reducing the rotational speed to 300 rpm proved more advantageous for the growth of recombinant *C. glutamicum.* In the later stages, increasing the rotational speed to 500 rpm was beneficial for ensuring adequate oxygen supply, which in turn supported HMW-heparosan production. Ultimately, the highest HMW-heparosan titer achieved was 7.02 g/L (Fig. 5B), with a high molecular weight of 801 kDa.

## Conclusion

In this work, we engineered *Corynebacterium glutamicum* to produce HMW-heparosan with high titer and satisfied molecular weight. HMW-heparosan synthase (PmHS2) expressed in *C. glutamicum* and successfully constructed the high- molecular weight HMW-heparosan synthesis pathway. The co-overexpression of key enzymes increased to 426.7 mg/L of HMW-heparosan. Then, the attenuation of extracellular polysaccharide biosynthesis and regulation of cell growth enhanced HMW-heparosan production, in which the overexpression of *whcD* and *PnkB* led to a 1.5-fold and 1.4-fold increase in cell density and consequently an increase HMW-heparosan titer to 1,057.2 mg/L. Additionally, relocation of the HMW-heparosan synthase on the cell membrane guided by secrete signal peptides and cell membrane display motifs increased the HMW-heparosan titer to 1.40 g/L. The engineered strain produces 7.02 g/L HMW-heparosan in fed-batch with 801 kDa. These results demonstrate combinatorial optimization of cell factories and the extracellular environment is efficacious and likely applicable for the production of other biopolymers. The intracellular accumulation of HMW-heparosan might intrinsically limit yield enhancement, and the lack of systematic analysis of its metabolic fluxes further might hinders precise pathway optimization. To systematically address these barriers, our next step will focus on integrating dynamic metabolic regulation with advanced fermentation strategies, thereby achieving higher titers while simultaneously enabling tailored molecular weight control.

## Electronic supplementary material

Below is the link to the electronic supplementary material.

Supplementary Material 1

## Data Availability

All data generated or analyzed during this study are included in this published article and its additional files. The authors are willing to provide any additional data and materials related to this research that may be requested for research purposes.
