# Supplementary material for: Coupling optimization of cell growth cycle and key enzyme membrane localization for enhanced synthesis of high molecular weight heparosan by Corynebacterium glutamicum
Source: Bioresour Bioprocess. 2025 Jun 17;12(1):61. doi: 10.1186/s40643-025-00899-0 (PMC12170474; doi:10.1186/s40643-025-00899-0)
Supplement: Supplementary file 1 — Supplementary Material 1 [file 40643_2025_899_MOESM1_ESM.docx]

**Supplementary Materials for:**

**Coupling optimization of cell growth cycle and key enzyme membrane localization for enhanced synthesis of high molecular weight heparosan by *Corynebacterium glutamicum*.**


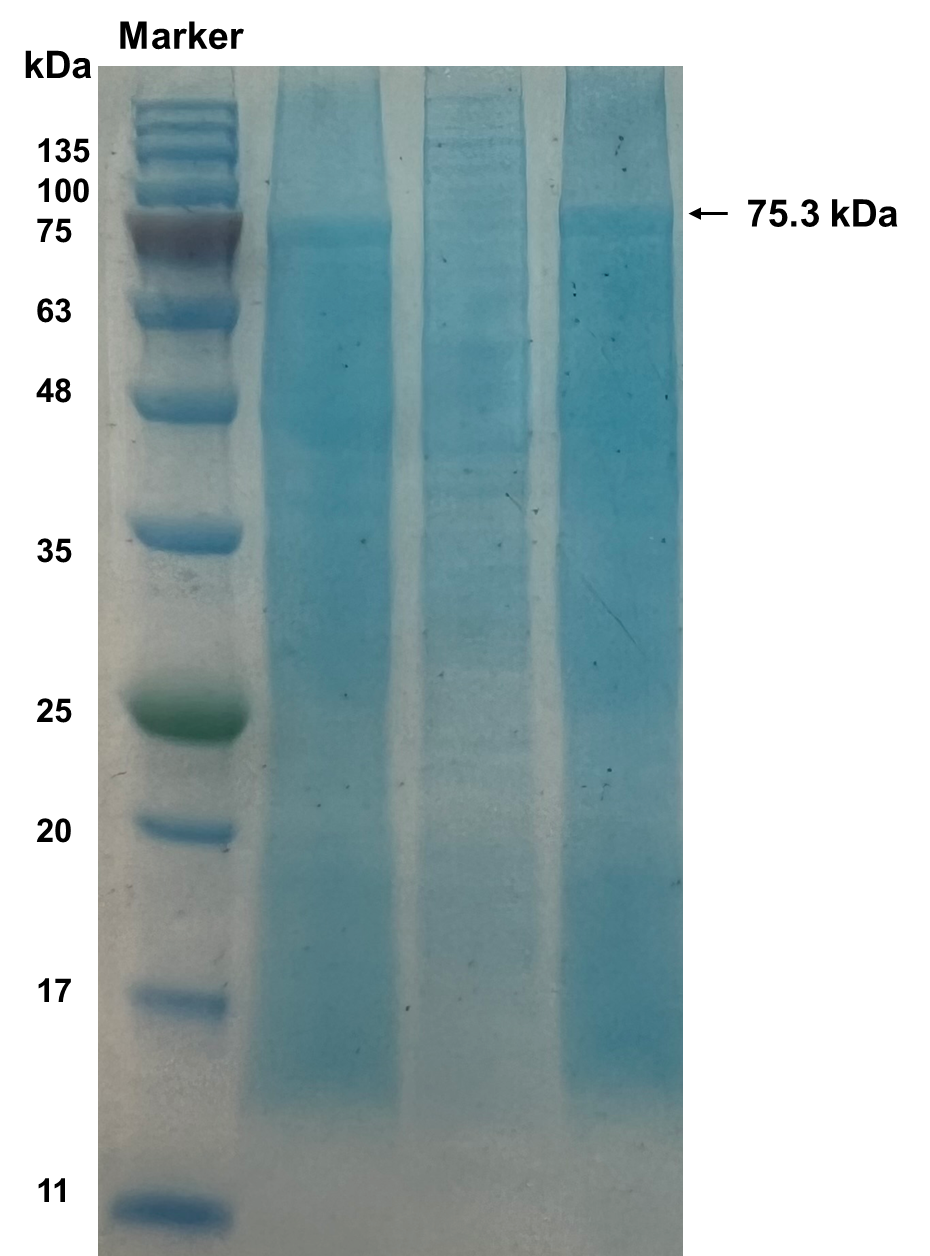


Figure S1 SDS-page analysis of PmHS2.


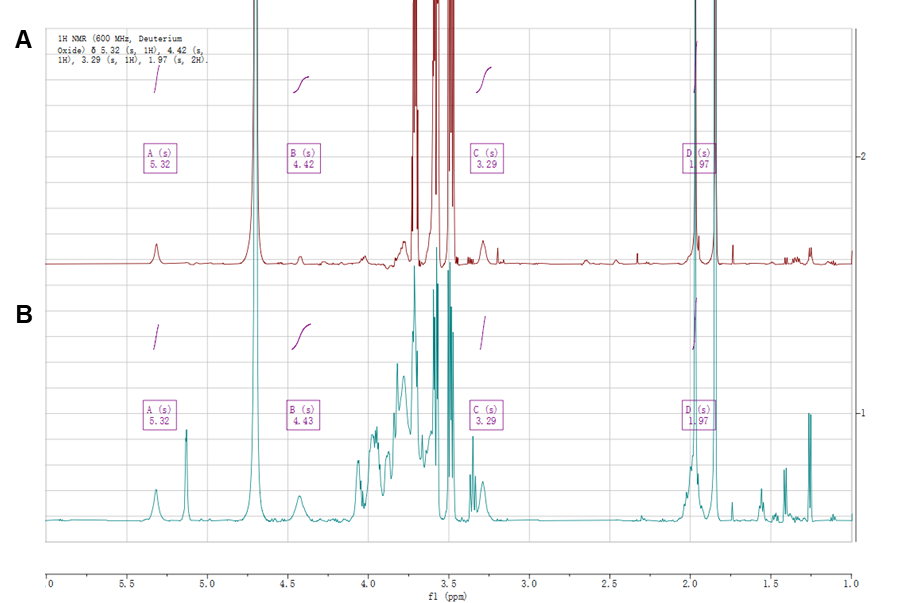


Figure S2 ^1^H NMR spectra of heparosan products from the engineered *C. glutamicu*m strain. A. the standard of heparosan. B. bioreactor cell pellet product

Figure S3 The SAX-HPLC anaylsis of HMW-heparosan disaccharides of ΔUA-GlcNAc


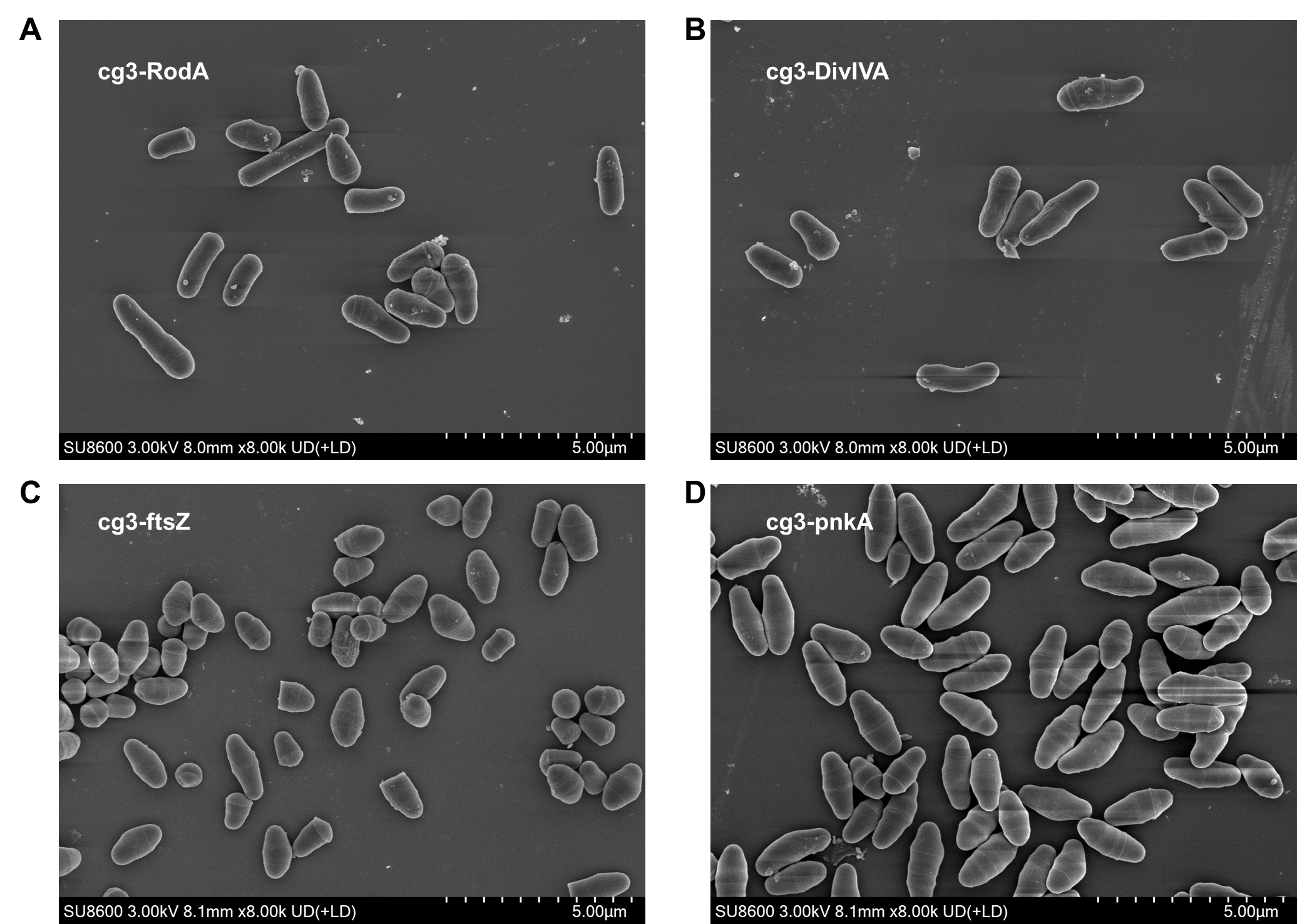


Figure S4 The cell morphology of cg3-RodA, DivIVA, FtsZ and PnkA.


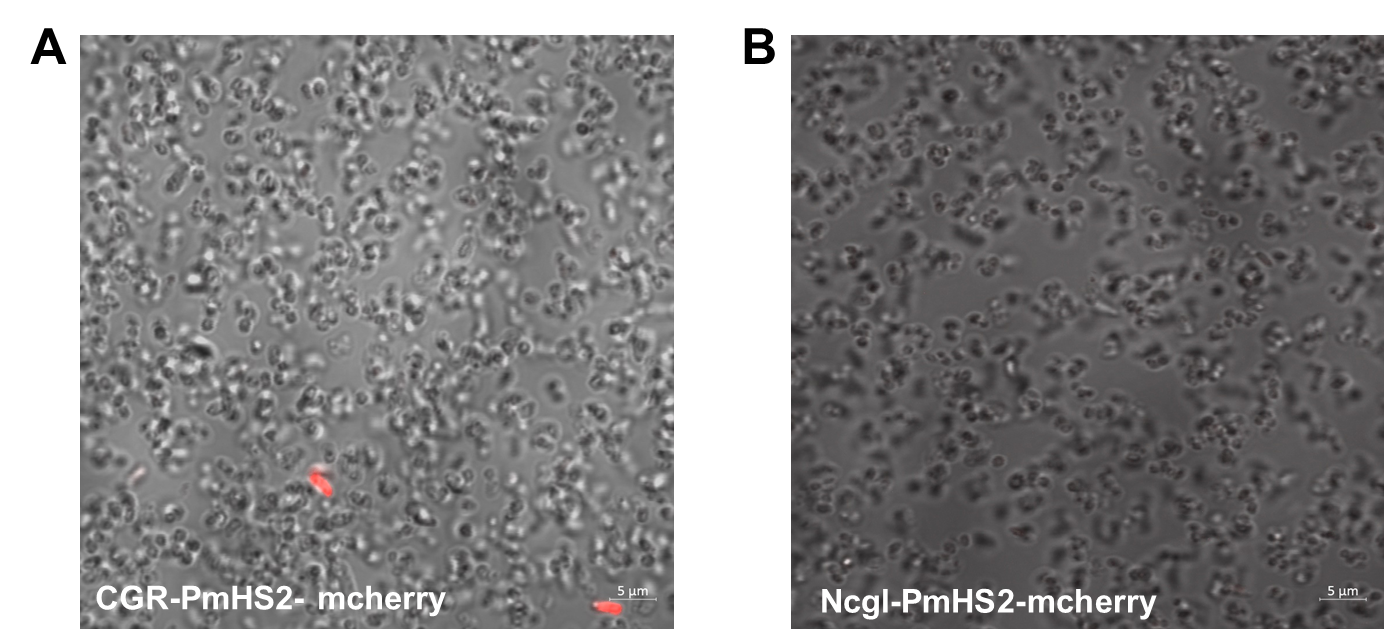


Figure S5 The CLSM results of membrane-located expression of PmHS2. A. The CLSM result of CGR-PmHS2-mchrerry. B. The CLSM result of Ncgl-PmHS2-mcherry.

Table S1 ^1^H NMR chemical shift assignments for heparosan products from the engineered *C. glutamicum*

|  | Proton | Chemical shift (ppm) |
| --- | --- | --- |
| A | GlcNAc, H1 | 5.32 |
| B | GlcA H1 | 4.43 |
| C | GlcA H2 | 3.32 |
| D | GlcNAc, CH3 | 1.97 |
